# Favorable long-term health-related quality of life after surgery for lumbar disc herniation in young adult patients

**DOI:** 10.1007/s00701-023-05522-9

**Published:** 2023-02-19

**Authors:** Miika Ilvesroiha, Johan Marjamaa, Jari Siironen, Seppo Koskinen, Anniina Koski-Palkén

**Affiliations:** 1 Department of Neurosurgery, Helsinki University Hospital, Helsinki, Finland; 2 Faculty of Medicine, University of Helsinki, Helsinki, Finland; 3 Finnish Institute for Health and Welfare, Helsinki, Finland

**Keywords:** Lumbar disc herniation, Microdiscectomy, Long-term outcome, Health-related quality of life

## Abstract

**Background:**

Lumbar disc herniation is often managed conservatively; nevertheless, surgical intervention can be required. Majority of patients experience a drastic relief of symptoms after surgery, but previous studies have reported that their health-related quality of life remains inferior compared to the general population for several years. There may be a major cumulative loss of health-related quality of life for young patients as they have long expected life ahead of them.

**Methods:**

A total of 526 eligible adult patients under the age of 40 underwent surgery for lumbar disc herniation from 1990 to 2005. Patients’ baseline characteristics were acquired by chart review to confirm eligibility to the study. Follow-up quality of life data was acquired by sending patients EQ-5D questionnaire at median 18 years after index surgery, and those 316 patients responding to the questionnaire (60%) were included in the study. Propensity score matching was utilized to match every study patient with two general population sample participants from a large Finnish population health study. Primary objective was to compare the quality of life to that of the control population. Secondary objective was to explore which patient characteristics lead to inferior outcome.

**Results:**

The mean EQ-index for the patient cohort was 0.86, while it was 0.84 for the age and gender–matched general population sample (difference 0.02, 95% CI − 0.0004 to 0.049). Within the patient cohort, an increasing number of lifetime lumbar surgeries was associated with progressively deteriorating EQ-index scores (*p* = 0.049) and longer duration of symptoms prior to the surgery correlated with lower score (*p* = 0.013).

**Conclusion:**

Patients who underwent surgery for lumbar disc herniation nearly two decades ago reported quality of life comparable to the age and gender–matched general population. However, patients who had undergone numerous lumbar surgeries had significantly worse outcome. Therefore, possible ways to prevent cumulation of lumbar surgeries could improve long-term health-related quality of life.

## Introduction

Lumbar disc herniation frequently presents with lower back pain accompanied with sciatica [5]. It has a naturally favorable clinical course, with spontaneous recovery occurring in up to 87% of cases within 3 months [24]. Sometimes surgical treatment is warranted if acute symptoms are intolerable or neurologically severe or agonizing pain continues for an extended time period. Early surgery within 6 to 12 weeks after onset of symptoms leads to faster short-term recovery compared to conservative treatment, but the difference diminishes in 1-year follow-up [16].

Patients undergoing surgery for lumbar disc herniation suffer from disability and drastically lowered health-related quality of life prior to surgery, while after surgery, the health-related quality of life improves rapidly during the first year [8, 12]. However, previous studies suggest that little or no improvement occurs during the second year [6], and no improvement between 2 and 7 years after surgery [21]. In these studies, the patients’ health-related quality of life remained lower than reported general population values at all follow-up time points. If the health-related quality of life remains reduced for an extended period, the disease burden is considerable, especially for young patients as they have long expected life ahead of them.

Previously, we reported the long-term outcome of an adult patient cohort who underwent surgery for lumbar disc herniation below the age of 40 years [18]. Our results showed that despite a 26% need for further surgery during the median 18-year follow-up period, the patients were highly satisfied with the outcome of their surgery, and they demonstrated a favorable employment status and reported Oswestry Disability Index scores similar to previously reported normative values.

In the present study, we evaluated the health-related quality of life of the patient cohort 18 years after they had undergone surgery for lumbar disc herniation. To our knowledge, no studies have reported over a 7-year outcome after surgery for lumbar disc herniation measured by health-related quality of life. The primary objective of the study was to compare the health-related quality of life of the patient cohort to that of an age and gender–matched Finnish population sample. The secondary objective was to discover which patient characteristics predispose the patient to worse health-related quality of life compared to the other patients.

## Methods

### Data collection

The study was conducted in the Department of Neurosurgery at Helsinki University Hospital. Helsinki University Hospital (HUS) is a publicly funded hospital in southern Finland, consisting of 23 hospitals or clinics. The ethical committee of HUS approved the study prior to its initiation.

The patient cohort included all 316 adult patients below the age of 40 years without history of previous lumbar spine surgery who underwent surgery for lumbar disc herniation at the department during the period from 1990 to 2005 and responded to health-related quality of life questionnaires. The medical records of all patients undergoing surgery for lumbar disc herniation within the years 1995–2005 were examined to identify candidates for the study (*n* = 615). Patients with history of previous lumbar surgery as well as patients whose symptoms were not caused by a lumbar disc herniation were excluded (*n* = 89). All patients had radiological confirmation of lumbar disc herniation prior to surgery, and it was conducted with a magnetic resonance imaging (MRI). At this point, 526 candidate patients were identified. In addition to confirming eligibility to the study, the medical records were utilized to collect patient characteristics such as age, body mass index, duration of symptoms, smoking, and possible further lumbar spine surgeries during the follow-up period.

These patients were contacted to gather long-term follow-up data. Contacting patients nearly two decades after surgery was possible due to the Finnish population register system which allowed us to request the patients’ current addresses from the Digital and Population Data Services Agency. Subsequently, the patients were sent information on the study, the informed consent form, and two questionnaires.

The first questionnaire was the EuroQol-5D-3L (EQ-5D-3L) questionnaire which is a frequently utilized instrument to measure health-related quality of life [4, 7]. In the EQ-5D-3L questionnaire, five dimensions of health (mobility, self-care, usual activities, pain/discomfort, depression/anxiety) are assessed on a 3-level scale (no problems, moderate problems, extreme problems). The answers can be converted in a single value, EQ-index, by utilizing valuation sets. The valuation set ranges from 0 (worst possible health) to 1 (best possible health) [15]. Additionally, the respondents report their self-perceived health with a visual analogue scale, EQ-VAS, ranging from 0 (worst imaginable health) to 100 (best imaginable health).

**Fig. 1:**
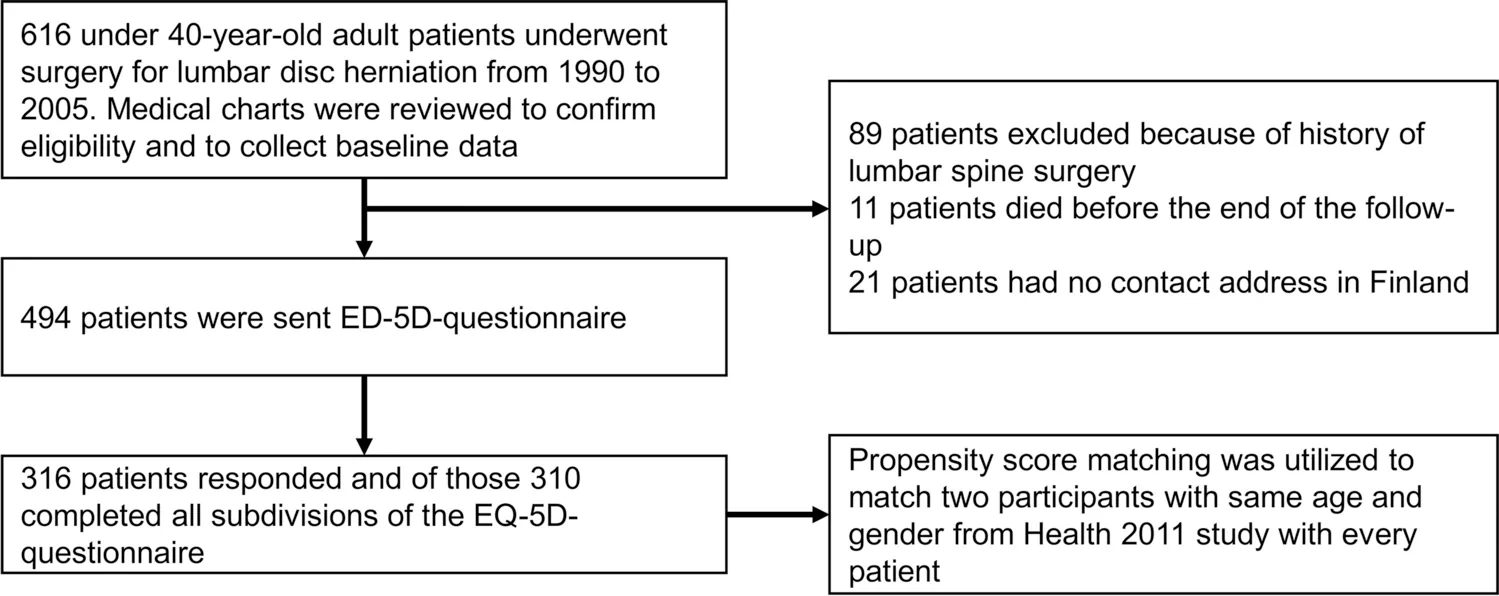
The data collection and propensity score matching protocol. In total, 615 adults under 40 years underwent surgery for lumbar disc herniation from 1990 to 2005. Of these patients, 89 were excluded because of history of lumbar surgery. The patients were contacted to collect follow-up data, and 316 patients responded. The patients who completed all parts of the EQ-5D questionnaire were matched with two same gender and age participants from Health 2011 study

The second questionnaire was a general questionnaire inquiring the patient’s smoking prior to the index surgery and nowadays, and possible further lumbar spine surgeries after the index surgery. The information on further lumbar surgeries was used to complement the data collected from the medical records. Non-responding patients were sent one reminder. Three hundred sixteen patients (60% of all candidates) responding to the questionnaires were included in the study. The data collection protocol is summarized in Fig. 1.

### Patient cohort

The total number of patients included in the patient cohort was 316, with a median follow-up of 19.1 (IQR 6.0) years (Table 1). The cohort included 171 males (54%) and 145 females (46%). The median age at the time of the index surgery was 33.5 years (IQR 7.4), and 28% of the patients were younger than 30 years at that time. At the time of the index surgery, the patients’ average body mass index (BMI) was 23.9 (SD 4.3) and 30% of the patients were smokers. The duration of symptoms prior to the index surgery was less than 6 months for 55% of the patients, 28% had symptoms for 6 to 12 months, and the symptoms had persisted for over 12 months for 17% of the patients. At the end of the follow-up period, 150 patients (27%) had undergone at least one additional lumbar surgery.

**Table 1:** Patient cohort characteristics

	Patients contacted, *N* (%)	Responders (*N*)	Responding rate (%)	*p* value^1^
All patients	526	316	60	
Follow-up time (years, median, IQR)	18.3 (6.5)	19.1 (6.0)		0.032
Gender				
Male	314 (60%)	171 (54%)	55	0.001
Female	212 (40%)	145 (46%)	68	
Age at baseline (median, IQR)	32.2 (7.6)	33.5 (7.4)		0.017
Under 30 years old during surgery	165 (31%)	88 (28%)	53	0.035
Age at follow-up (median, IQR)	53.0 (8.9)	53.6 (8.6)		0.004
Smoking at the time of the surgery^2^				
Yes	133 (36%)	65 (30%)	49	0.006
No	238 (64%)	152 (70%)	64	
Body mass index at the time of the surgery^2^				
Mean (SD)	24.3 (4.2)	23.9 (4.3)		0.001
< 18.5	7 (1.4%)	5 (1.7%)	71	
18.5–25	291 (58%)	188 (62%)	65	
25–30	172 (34%)	98 (32%)	57	
> 30	31 (6.2%)	12 (4.0%)	39	
Duration of symptoms prior to surgery				
1 to 6 months	292 (56%)	174 (55%)	60	0.74
6 to 12 months	139 (26%)	85 (28%)	63	
Over 12 months	95 (18%)	55 (17%)	58	
Further lumbar surgery during follow-up period				
Yes	150 (29%)	85 (27%)	57	0.33
No	376 (71%)	231 (73%)	61	
Number of further lumbar spine surgeries				
0	376 (71%)	231 (73%)	61	0.09
1	107 (20%)	67 (21%)	63	
2	26 (4.9%)	10 (3.2%)	62	
3 or more	17 (3.2%)	8 (2.5%)	47	

^1^Statistical significance of the difference between the responders and non-responders in the characteristic

^2^Information on smoking status prior to surgery was available for 371 patients and information on body mass index for 501 patients

Of the initially identified 526 candidate patients, 316 (60%) returned the EQ-5D-3L questionnaire, and 310 patients completed every part of the questionnaire. The patients who completed the follow-up questionnaire had significantly different characteristics than the non-responding candidate patients. They were older both at the time of the surgery (*p* = 0.02) and at the follow-up (*p* = 0.004), more often female (*p* = 0.001), and non-smokers at baseline (*p* = 0.006) and had lower baseline BMI (*p* = 0.001) (Table 1).

### General population sample and propensity score matching

A general population sample was used to compare patient cohort’s health-related quality of life to that of the general population. General population health surveys are conducted frequently in Finland by the Finnish National Institute for Health and Welfare (THL). THL carried out a large population study, Health 2011 Survey, in 2011 [1]. The sample of the Health 2011 Survey included 8135 individuals aged 29 years or over representing the Finnish adult population, and 5903 persons participated in the comprehensive study protocol, including a thorough health examination, laboratory tests, and multiple questionnaires. One of the questionnaires was EQ-5D-3L, allowing us to compare the health-related quality of life between our patient cohort and a general population sample.

To form a general population sample comparable to our patient cohort, propensity score matching was conducted to create an age and gender–matched general population sample [2]. Each member of our patient cohort who had completed every part of the EQ-5D-3L questionnaire (*n* = 310) was matched with two participants from the Health 2011 study population according to age and sex. The matching was done with the statistical software R (R Core Team, 2021) by utilizing the extension package MatchIt [11]. After propensity score matching, the matched general population sample consisted of 620 participants with the same median age (54 years) and gender distribution (46% female) as in the patient cohort.

### Statistical methods

All statistical tests were conducted with R, version 4.0.5. A *t* test was done to analyze the difference in means of independent continuous variables between two dependent categorical variables, and the Levene test was run prior to the *t* test to choose the correct assumption of equality of variance for the *t* test. One-way ANOVA was utilized if the number of categorical variables was higher than 2. Ordinal variables were analyzed with the Mann–Whitney *U* and Kruskal–Wallis tests. The correlation between two variables was measured with the Spearman rank correlation test. The Fisher exact test was used to analyze the 2 × 2 contingency tables. A *p* value less than 0.05 was considered significant for all statistical tests.

## Results

### EQ-5D-3L dimensions

**Fig. 2:**
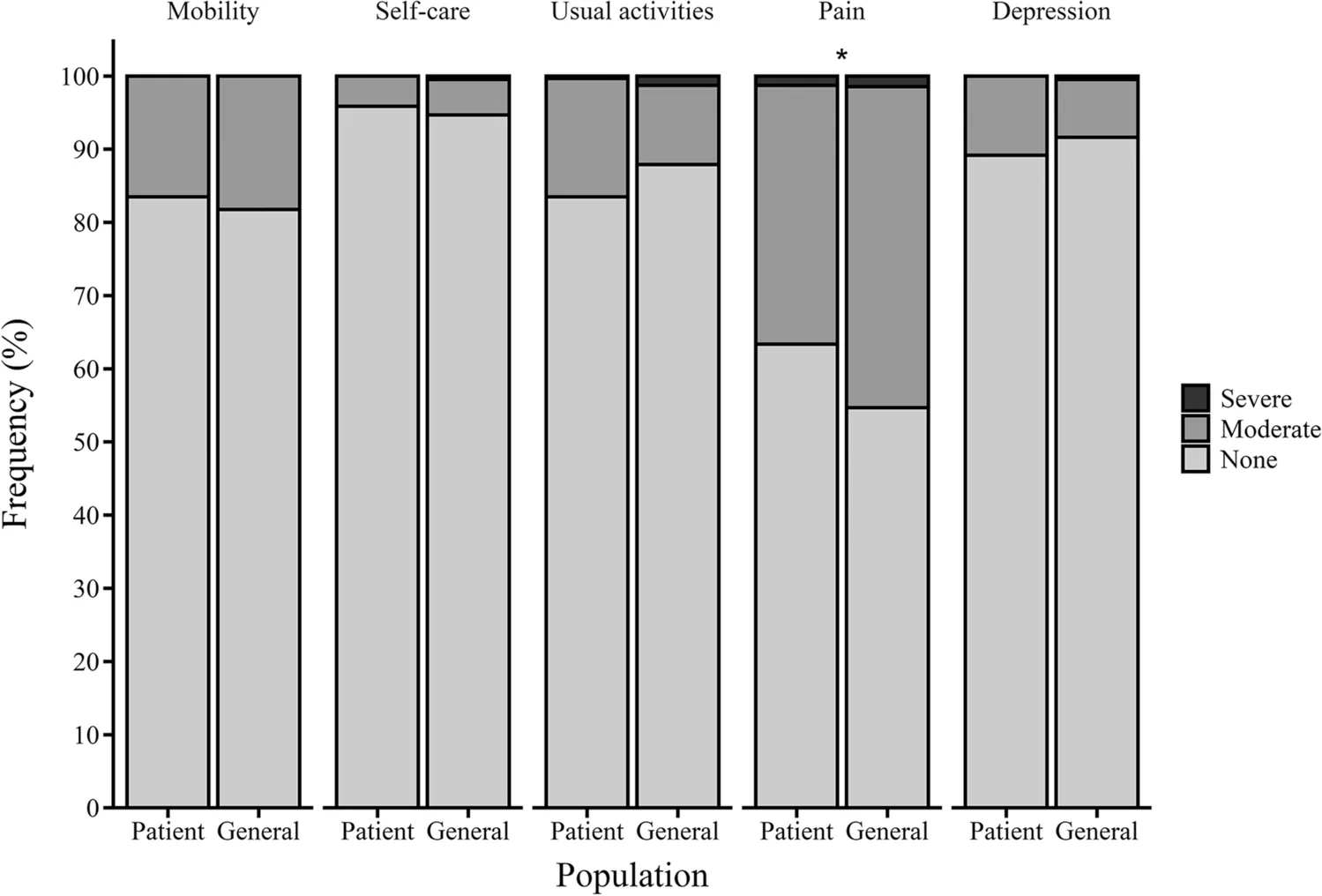
Patient cohort and matched general population cohort responses to the EQ-5D questionnaire. The patient cohort included 316 patients while the general population sample size included 620 age and gender–matched respondents. In the questionnaire, the degree of problems experienced by the respondent on each aspect of health is reported on a 3-level scale (no problems, moderate problems, severe problems). The difference between the groups was analyzed with the Mann–Whitney *U* test. *p < 0.05

The patient cohort reported mobility problems as often as the general population sample (*p* = 0.51) (Fig. 2). Within the patient cohort, patients who were overweight at the time of the surgery reported more problems (*p* = 0.004) (Table 2). Additionally, an increasing number of lifetime lumbar spine surgeries (*p* = 0.002) and longer duration of symptoms (*p* = 0.013) prior to the index surgery correlated with more frequent problems in mobility. Other baseline characteristics, gender (*p* = 0.71), age (*p* = 0.61), and smoking (*p* = 0.64), were not significantly associated with mobility.

**Table 2:** Patient responses to the EQ-5D-3L questionnaire by baseline characteristics of subgroups

	Mobility			Self-care			Usual activities			Pain/discomfort			Depression/anxiety		
	No	Some	Severe	No	Some	Severe	No	Some	Severe	No	Some	Severe	No	Some	Severe
All patients (*n* = 316) (%)	83	17	0	96	4.1	0	83	16	0.3	63	35	1.3	89	11	0
Gender								**			*				
Male (*n* = 171) (%)	84	16	0	97	2.9	0	89	11	0.7	69	29	1.2	90	10	0
Female (*n* = 145) (%)	83	17	0	94	5.5	0	77	23	0	56	42	1.4	88	12	0
Age^1^															
Over 30 (*n* = 228) (%)	83	17	0	96	4.4	0	84	15	0.4	63	35	1.3	88	12	0
Under 30 (*n* = 88) (%)	85	15	0	97	3.4	0	82	18	0	63	36	1.1	92	8	0
Smoking^1^															
Yes (*n* = 96) (%)	82	18	0	97	3.1	0	81	19	0	57	43	0	85	15	0
No (*n* = 210) (%)	84	16	0	95	4.7	0	83	16	0.5	66	32	1.9	92	8	0
Body mass index^1^		**													
Over 25 (*n* = 110) (%)	75	25	0	94	6.3	0	80	19	0.9	58	40	1.8	90	10	0
Under 25 (*n* = 193) (%)	88	12	0	97	2.6	0	85	15	0	65	34	0.5	89	11	0
Duration of symptoms^1^		*			*			*			**				
Under 6 months (*n* = 174) (%)	88	12	0	98	2.3	0	87	13	0	79	29	1.7	90	10	0
6 to 12 months (*n* = 87) (%)	80	20	0	95	4.6	0	83	17	0	61	39	0	92	8	0
Over 12 months (*n* = 55) (%)	75	25	0	91	9.1	0	73	25	1.8	49	49	1.8	83	17	0
Further lumbar surgeries^2^		**			**			*			*				
0 (*n* = 231) (%)	87	13	0	98	2.2	0	86	13	0.4	67	33	0	90	10	0
1 (*n* = 67) (%)	75	25	0	93	7.5	0	79	21	0	55	42	3.0	88	12	0
2 or more (*n* = 18) (%)	67	33	0	83	17	0	67	33	0	56	33	11	83	17	0

The statistical significance was tested with the Mann–Whitney *U* test or Spearman rank correlation test

^*^p < 0.05; **p < 0.01

^1^Age, smoking status, and body mass index during the index surgery and duration of symptoms prior to it

^2^The number of patients who had undergone further lumbar surgeries at the end of the follow-up period

Similarly, there was no overall difference in the self-care dimension of EQ-5D between the patient cohort and general population sample (*p* = 0.41, Fig. 2), but patients with a higher number of lumbar surgeries (*p* = 0.002) and longer duration of symptoms experienced more problems (*p* = 0.037). Age (*p* = 0.69), gender (*p* = 0.25), BMI (*p* = 0.11), and smoking at the time of the surgery (*p* = 0.51) did not have an impact on self-care dimension scores (Table 2).

On the other hand, in usual activities, the patient cohort had a trend towards more problems than the general population sample (*p* = 0.074, Fig. 2). Similarly again, a higher number of lumbar surgeries (*p* = 0.026 and longer duration of symptoms (*p* = 0.017) were correlated with more frequent problems. Furthermore, females reported more problems (*p* = 0.006), while age (*p* = 0.63), smoking (*p* = 0.61), and BMI (*p* = 0.24) at baseline had no effect on the outcome (Table 2).

In the pain and discomfort dimension, fewer patients reported problems compared to the general population sample participants (*p* = 0.012, Fig. 2). An increasing number of lumbar spine surgeries correlated with an inferior outcome also in this dimension (*p* = 0.030), as well as a longer duration of symptoms prior to surgery (*p* = 0.010). Age (*p* = 0.98), BMI (*p* = 0.19), and smoking (*p* = 0.17) at baseline did not affect the results, but women (*p* = 0.017) reported more problems than men (Table 2).

In the depression and anxiety dimension, the patient cohort reported as much problems as the general population sample (*p* = 0.23, Fig. 2). All baseline characteristics were insignificant as risk factors in this dimension (Table 2).

### EQ-index

**Fig. 3:**
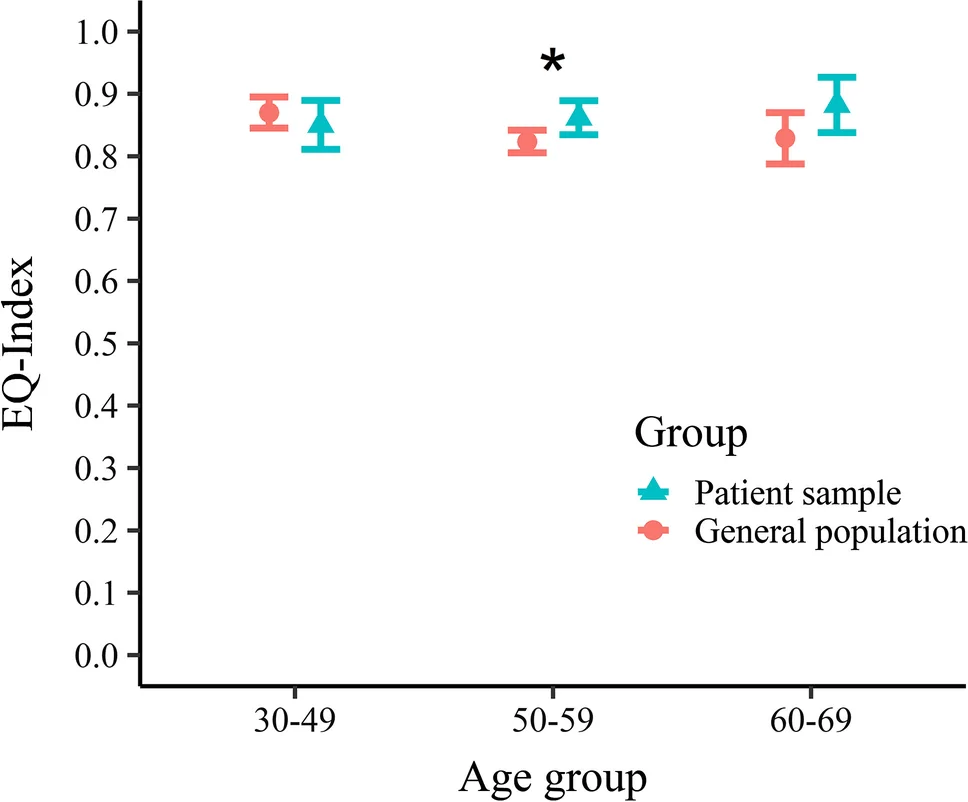
EQ-index scores in different age groups in the patient cohort and in the general population sample. Blue triangles show the mean score for the patient cohort (*n* = 310) and red dots that of the general population sample (*n* = 620). Error bars display the 95% confidence interval for the values. Mean scores in the patient cohort and in the general population sample in age groups were as follows: 30–49 (0.85 vs. 0.87, *p* = 0.39), 50–59 (0.86 vs. 0.82, *p* = 0.021), and 60–69 (0.88 vs. 0.83, *p* = 0.086). Student’s *t* test was conducted to calculate the *p* value. *p < 0.05

The mean EQ-index score in the patient cohort was 0.86, while it was 0.84 in the general population sample (*p* = 0.054) (Table 3). The patient cohort had statistically significantly slightly higher scores in the age group 50–59 years (0.86 vs. 0.82, *p* = 0.021), but there were no significant differences in the other age groups (Fig. 3).

**Table 3:** EQ-index scores

	Number	Mean score	*p* value^1^
All patients	310	0.86	
General population sample	620	0.84	0.054
Gender			
Male	167	0.88	0.021
Female	143	0.84	
Age at the time of the surgery			
Over 30	223	0.86	0.64
Under 30	87	0.87	
Smoking at the time of the surgery			
Yes	95	0.84	0.18
No	205	0.87	
Body mass index			
Over 25	107	0.84	0.33
Under 25	190	0.87	
Duration of symptoms prior to surgery			
Under 6 months	167	0.88	0.013
6 to 12 months	87	0.85	
Over 12 months	54	0.81	
Further lumbar spine surgeries			
0	228	0.87	0.049
1	64	0.83	
2 or more	18	0.80	

^1^Student’s *t* test or Spearman rank correlation test

A higher number of lifetime lumbar surgeries correlated with lower EQ-index score (*p* = 0.049) (Table 3). Patients without further surgery reported a mean score of 0.88; for those with one additional surgery, it was 0.83; and the mean score was 0.80 for those with two or more further surgeries.

Furthermore, prolongation of symptoms prior to the index surgery was correlated with progressively worsening EQ-index score (*p* = 0.013). The mean scores for different symptom duration groups were as follows: under 6 months, 0.88; 6 to 12 months, 0.85; and over 12 months, 0.81. Additionally, males reported higher scores than females (0.88 vs. 0.84, *p* = 0.021) while the other baseline characteristics, age (*p* = 0.64), smoking (*p* = 0.18), and BMI (*p* = 0.33), had no effect on the scores.

#### EQ-VAS

EQ-VAS scores were available for the patient cohort but not in the general population sample. The mean EQ-VAS score for patients was 81.6 (Table 4). Patients who had symptoms for less than 6 months and 6 to 12 months reported similar scores (82.3 vs. 83.5), while patients suffering from symptoms for over 12 months reported a significantly lower score of 77.8 compared to patients with shorter duration of symptoms (*p* = 0.048). A higher number of lumbar surgeries correlated with worse self-perceived health; the mean EQ-VAS score for patients without further surgery was 82.7, it was 80.3 for patients with one additional surgery, and for patients with at least two further surgeries, it was 76.2 (*p* = 0.070). Other baseline characteristics, age (*p* = 0.25), gender (*p* = 0.068), BMI (*p* = 0.30), and smoking (*p* = 0.39), were not significantly associated with EQ-VAS.

**Table 4:** Patient responses to EQ-VAS

	Number	Mean score	*p* value^1^
All patients	311	81.9	
Gender			
Male	168	83.2	0.068
Female	143	80.3	
Age at the time of the surgery			
Over 30	225	81.3	0.25
Under 30	86	83.3	
Smoking at the time of the surgery			
Yes	96	81.0	0.39
No	205	82.5	
Body mass index			
Over 25	107	81.0	0.30
Under 25	191	82.7	
Duration of symptoms prior to surgery^2^			
Under 6 months	171	82.3	0.048
6 to 12 months	86	83.5	
Over 12 months	54	77.8	
Further lumbar surgeries			
0	228	82.8	0.070^3^
1	66	80.3	
2 or more	17	76.2	

^1^Student’s *t* test unless otherwise stated

^2^Tukey HSD post hoc analysis: under 6 months vs. over 12 months (*p* = 0.088) and 6 to 12 months vs. over 12 months (*p* = 0.046)

^3^Spearman rank correlation test

## Discussion

The results on long-term health-related quality of life after surgery for lumbar disc herniation have been sparse, and the few reports have indicated that the patients have impaired health-related quality of life even years after surgery. To our best knowledge, this study is the first one to present results indicating that long-term health-related quality of life after surgery for lumbar disc herniation is similar compared to the general population.

This is demonstrated by the patients reporting EQ-index scores comparable to an age and gender–matched general population sample. The mean EQ-index score in the patient cohort was even statistically significantly slightly better that that in the age and gender–matched general population sample (0.86 vs. 0.84, *p* = 0.02 for difference). However, as the minimal clinically important difference (MCID) for EQ-5D index score has been proposed to be 0.07 [25], there was no clinically significant difference between these groups. Nevertheless, these results demonstrate that health-related quality of life of the patients is not worse compared to the general population in long-term follow-up.

Previous studies have shown that patients experience quality of life inferior to the normative values 1 year [8, 12], 2 years [6, 10], and even 7 years after surgery for lumbar disc herniation [21]. This study suggests that the health-related quality of life eventually reaches normative values. However, in the earlier studies, the average age of the patients was in early 40s compared to early 30s in our study. Furthermore, some earlier studies [12, 21] included patients who underwent a macrodiscectomy while our study cohort only included patients who underwent a microdiscectomy. In the study by Fisher et al. [8], the patient cohort had an average duration of symptoms of 51 weeks before surgery which may affect the results as long duration of symptoms has a negative influence on the long-term outcome [20]. These differences may limit the comparability of our results with the previous studies.

We observed a significant correlation between an increasing number of further lumbar surgeries and deteriorating quality of life and a clinically significant difference in quality of life when comparing patients without any further surgery and patients with at least two additionally surgeries (0.87 vs. 0.80). This is in accordance with earlier studies which have reported lower EQ-index scores [9, 14] and lower SF-36 bodily pain and physical function scores [13] for patients who underwent further lumbar spine surgery during the follow-up period. Additionally, Lubelski et al. [14] found that patients who underwent third surgery after revision discectomy had drastically impaired health-related quality of life. Nonetheless, EQ-index of 0.80 can be regarded as a good outcome as the general population value was 0.84.

Furthermore, patients whose symptoms prior to the index surgery exceeded 12 months reported worse quality of life than the other patients even nearly two decades after the index surgery. Their mean EQ-index score was 0.80, while it was 0.87 for patients whose symptom duration was less than 6 months and 0.83 for patients with the symptom duration between 6 and 12 months. The correlation between lengthening of symptoms and lowering EQ-index was statistically significant, but the difference is clinically significant only between the patients at the ends of the spectrum. This concurs with an earlier study which reported that, 1 year after surgery, patients who had symptoms for over 6 months reported worse EQ-index scores than patients with a shorter symptom duration [12]. Regardless of the chosen treatment, prolonged symptoms lead to inferior outcome, as in a 4-year observational follow-up study the symptom duration exceeding 6 months was associated with inferior SF-36 bodily pain and physical component scores with both treatment options [17]. However, a randomized trial reported that for patients who had symptoms for over 4 months, surgery led to better recovery from leg pain in 6-month follow-up [3].

The strength of the present study was the reliable conduction of the long-term follow-up which was achieved due to two factors. First, the HUS hospital district serves a population base of over 1 million people and its 23 hospitals have a common system of electronic medical records. This provided a reliable collection of patient history data from the medical records. Second, the Finnish personal register system allowed us to contact all patients still living in Finland even decades after the index surgery. This gave all patients an equal opportunity to participate in the study, minimizing selection bias.

The weakness of the study is that 60% of patients responded to the questionnaires 19 years after the index surgery. Wang et al. [26] reported that the response rate decays with time and 61% is the average rate after 5 to 10 years for cohort studies; hence, this study had reasonable response rate compared to previous studies. Nevertheless, the fairly high number of non-responders could affect the generalizability of our results. We did observe that the responding patients were older and more likely female and had lower BMI. Similar observations have been previously reported in Finnish population studies [23]. We did not account for education level in our study which could affect the results as people with higher education level respond more frequently [23] and report higher quality of life [19]. However, similar responding biases influenced the collection of general population sample (Health 2011), and it is therefore likely that our results provide a reliable picture of the differences in health-related quality of life between the patient cohort and the general population. Furthermore, two previous studies have reported that the short-term outcome after spine surgery is similar for responders and non-responders [6, 22].

## Conclusions

In conclusion, the results show that patients who underwent surgery for lumbar disc herniation as young adults reported health-related quality of life comparable to the general population nearly two decades after surgery. However, patients who had undergone repeated lumbar spine surgery experienced significantly inferior health-related quality of life compared to the other patients. This finding highlights the need to discover ways to prevent the cumulation of lumbar surgeries. Possible ways could include identifying the patients who have high risk for further surgery, correct patient selection for surgery, optimal surgical treatment, and postsurgical rehabilitation.

## Data Availability

The datasets generated during and/or analyzed during the current study are available from the corresponding author on reasonable request.

## References

[1] Annamari Lundqvist and Tomi Mäki-Opas, eds. Health 2011 survey – methods. The National Institute for Health and Welfare (THL). http://www.julkari.fi/handle/10024/130780. Accessed 5 Apr 2022

[2] Austin PC (2011) An introduction to propensity score methods for reducing the effects of confounding in observational studies. Multivariate Behav Res 46(3):399–424 21818162 10.1080/00273171.2011.568786 PMC3144483

[3] Bailey CS, Rasoulinejad P, Taylor D, Sequeira K, Miller T, Watson J et al (2020) Surgery versus conservative care for persistent sciatica lasting 4 to 12 months. N Engl J Med 382(12):1093–1102 32187469 10.1056/NEJMoa1912658

[4] Brooks R (1996) EuroQol: the current state of play. Health Policy 37(1):53–72 10158943 10.1016/0168-8510(96)00822-6

[5] Deyo RA, Mirza SK (2016) Herniated lumbar intervertebral disk. N Engl J Med 374(18):1763–1772 27144851 10.1056/NEJMcp1512658

[6] Elkan P, Lagerbäck T, Möller H, Gerdhem P (2018) Response rate does not affect patient-reported outcome after lumbar discectomy. Eur Spine J 27(7):1538–1546 29523985 10.1007/s00586-018-5541-0

[7] EuroQol Group (1990) EuroQol–a new facility for the measurement of health-related quality of life. Health Policy 16(3):199–208 10109801 10.1016/0168-8510(90)90421-9

[8] Fisher C, Noonan V, Bishop P, Boyd M, Fairholm D, Wing P et al (2004) Outcome evaluation of the operative management of lumbar disc herniation causing sciatica. J Neurosurg 100(4 Suppl Spine):317–24 15070138 10.3171/spi.2004.100.4.0317

[9] Fritzell P, Knutsson B, Sanden B, Strömqvist B, Hägg O (2015) Recurrent versus primary lumbar disc herniation surgery: patient-reported outcomes in the Swedish Spine Register Swespine. Clin Orthop Relat Res 473(6):1978–1984 24711131 10.1007/s11999-014-3596-8 PMC4418986

[10] Guilfoyle MR, Ganesan D, Seeley H, Laing RJ (2007) Prospective study of outcomes in lumbar discectomy. Br J Neurosurg 21(4):389–395 17676460 10.1080/02688690701477310

[11] Ho D, Imai K, King G, Stuart E (2011) MatchIt: nonparametric preprocessing for parametric causal inference. J Stat Softw 42(8):1–28. 10.18637/jss.v042.i08

[12] Jansson KA, Németh G, Granath F, Jönsson B, Blomqvist P (2005) Health-related quality of life in patients before and after surgery for a herniated lumbar disc. J Bone Joint Surg Br 87(7):959–964 15972911 10.1302/0301-620X.87B7.16240

[13] Leven D, Passias PG, Errico TJ, Lafage V, Bianco K, Lee A et al (2015) Risk factors for reoperation in patients treated surgically for intervertebral disc herniation: a subanalysis of eight-year SPORT data. J Bone Joint Surg Am 97(16):1316–1325 26290082 10.2106/JBJS.N.01287 PMC5480260

[14] Lubelski D, Senol N, Silverstein MP, Alvin MD, Benzel EC, Mroz TE, Schlenk R (2015) Quality of life outcomes after revision lumbar discectomy. J Neurosurg Spine 22(2):173–178. 10.3171/2014.10.SPINE14359 25478822 10.3171/2014.10.SPINE14359

[15] Ohinmaa A, Sintonen H (1999) Inconsistencies and modelling of the Finnish EuroQol (EQ-5D) preference values. In: Greiner W, von der Schulenburg JM Graf, Piercy J (eds) EuroQol plenary meeting, Hanover 1998, 1–2 October. Uni-Verlag Witte, Hanover, pp 57–74

[16] Peul WC, van Houwelingen HC, van den Hout WB, Brand R, Eekhof JA, Tans JT et al (2007) Surgery versus prolonged conservative treatment for sciatica. N Engl J Med 356(22):2245–2256 17538084 10.1056/NEJMoa064039

[17] Rihn JA, Hilibrand AS, Radcliff K, Kurd M, Lurie J, Blood E et al (2011) Duration of symptoms resulting from lumbar disc herniation: effect on treatment outcomes: analysis of the Spine Patient Outcomes Research Trial (SPORT). J Bone Joint Surg Am 93(20):1906–1914 22012528 10.2106/JBJS.J.00878 PMC5515548

[18] Roiha M, Marjamaa J, Siironen J, Koski-Palkén A (2022) Favorable long-term outcome in young adults undergoing surgery for lumbar disc herniation. Acta Neurochir (Wien). 10.1007/s00701-022-05375-8 36205789 10.1007/s00701-022-05375-8 PMC9705424

[19] Saarni SI, Härkänen T, Sintonen H, Suvisaari J, Koskinen S, Aromaa A et al (2006) The impact of 29 chronic conditions on health-related quality of life: a general population survey in Finland using 15D and EQ-5D. Qual Life Res 15(8):1403–1414. 10.1007/s11136-006-0020-1 16960751 10.1007/s11136-006-0020-1

[20] Silverplats K, Lind B, Zoëga B, Halldin K, Rutberg L, Gellerstedt M et al (2010) Clinical factors of importance for outcome after lumbar disc herniation surgery: long-term follow-up. Eur Spine J 19(9):1459–67. 10.1007/s00586-010-1433-7 20512513 10.1007/s00586-010-1433-7 PMC2989281

[21] Silverplats K, Lind B, Zoega B, Halldin K, Gellerstedt M, Rutberg L et al (2011) Health-related quality of life in patients with surgically treated lumbar disc herniation: 2- and 7-year follow-up of 117 patients. Acta Orthop 82(2):198–203 21434763 10.3109/17453674.2011.566136 PMC3235291

[22] Solberg TK, Sørlie A, Sjaavik K, Nygaard ØP, Ingebrigtsen T (2011) Would loss to follow-up bias the outcome evaluation of patients operated for degenerative disorders of the lumbar spine? Acta Orthop 82(1):56–63 21189113 10.3109/17453674.2010.548024 PMC3229998

[23] Tolonen H, Helakorpi S, Talala K, Helasoja V, Martelin T, Prättälä R (2006) 25-year trends and socio-demographic differences in response rates: Finnish adult health behaviour survey. Eur J Epidemiol 21(6):409–415 16804763 10.1007/s10654-006-9019-8

[24] Vroomen PC, de Krom MC, Wilmink JT, Kester AD, Knottnerus JA (1999) Lack of effectiveness of bed rest for sciatica. N Engl J Med 340(6):418–423 9971865 10.1056/NEJM199902113400602

[25] Walters SJ, Brazier JE (2005) Comparison of the minimally important difference for two health state utility measures: EQ-5D and SF-6D. Qual Life Res 14(6):1523–1532. 10.1007/s11136-004-7713-0 16110932 10.1007/s11136-004-7713-0

[26] Wang K, Eftang CN, Jakobsen RB, Årøen A (2020) Review of response rates over time in registry-based studies using patient-reported outcome measures. BMJ Open 10(8):e030808. 10.1136/bmjopen-2019-030808 32764078 10.1136/bmjopen-2019-030808 PMC7412618

